# Supplementary figures and images for: Characteristics of microRNAs in Skeletal Muscle of Intrauterine Growth-Restricted Pigs
Source: Genes (Basel). 2023 Jun 28;14(7):1372. doi: 10.3390/genes14071372 (PMC10379088; doi:10.3390/genes14071372)

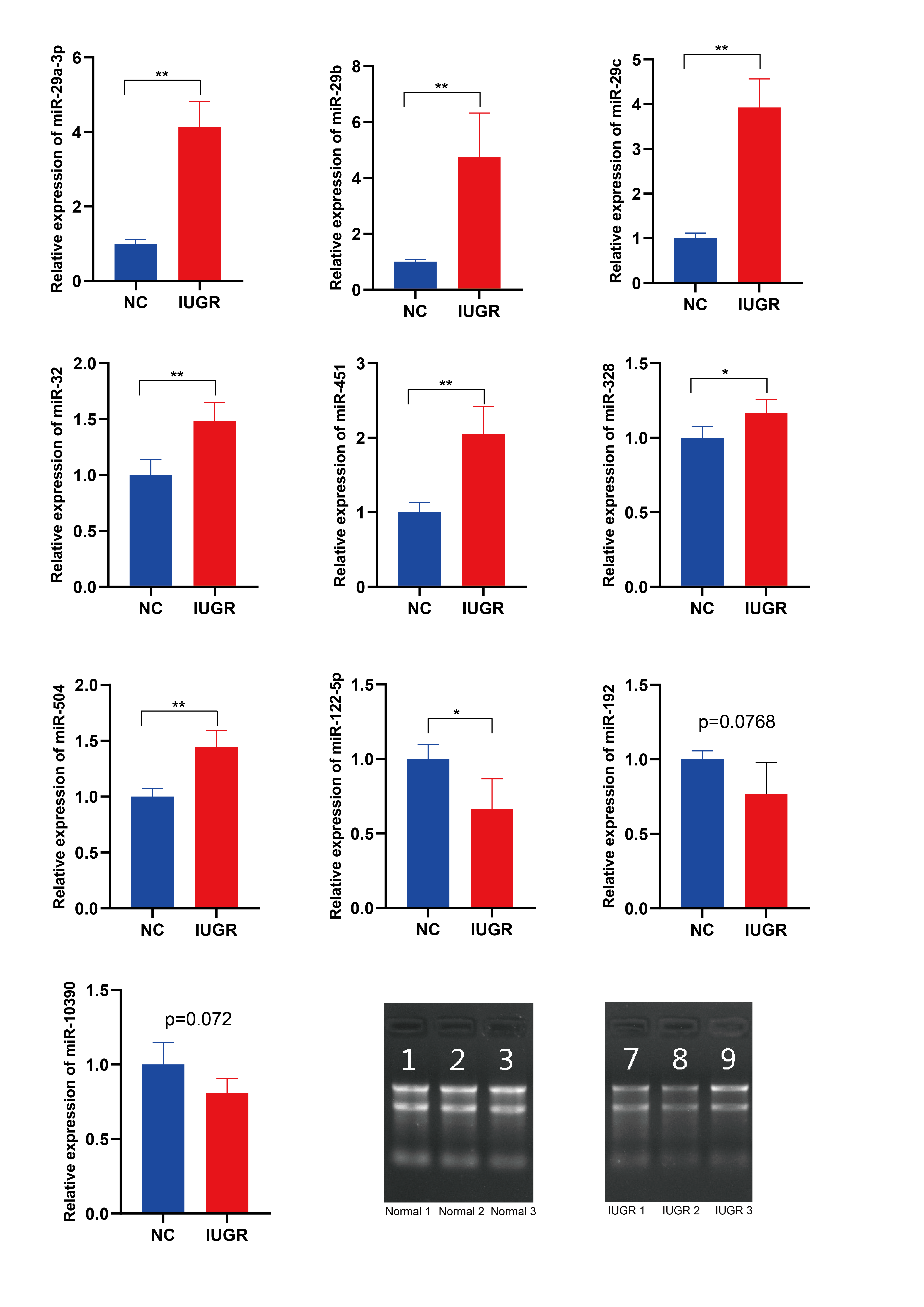

Supplement: Supplementary file 1 [file genes-14-01372-s001.zip › S/Figure S1.png]
